# Draft Genome Sequence of Faecalimonas umbilicata JCM 30896^T^, an Acetate-Producing Bacterium Isolated from Human Feces

**DOI:** 10.1128/MRA.01091-18

**Published:** 2018-09-06

**Authors:** Mitsuo Sakamoto, Nao Ikeyama, Masahiro Yuki, Moriya Ohkuma

**Affiliations:** a Microbe Division/Japan Collection of Microorganisms, RIKEN BioResource Research Center, Tsukuba, Japan; b PRIME, Japan Agency for Medical Research and Development (AMED), Tsukuba, Japan; Queens College

## Abstract

Here, we report the draft genome sequence of Faecalimonas umbilicata JCM 30896^T^, an acetate-producing bacterium isolated from human feces. The genomic analysis reveals genes for acetate and vitamin B_12_ synthesis and will facilitate the study of the role of this strain in the human gut.

## ANNOUNCEMENT

Acetate is one of the metabolic end products of anaerobic fermentation. Butyrate-producing bacteria, such as Faecalibacterium prausnitzii and *Roseburia* spp., can be net utilizers of acetate (1). The butyrate formed by the above-mentioned acetate-consumers has important roles in colonic health (1). Recently, we isolated a new acetate-producing bacterium, Faecalimonas umbilicata JCM 30896^T^, from a fecal sample from a healthy Japanese man (2). This species is a member of the family *Lachnospiraceae*. Anaerobic bacteria affiliated with the family *Lachnospiraceae* make up the majority of highly prevalent bacteria in the human intestinal tract (3). We analyzed the draft genome sequence of F. umbilicata JCM 30896^T^ to improve our understanding of the physiology and potential health contribution of this strain in the human gut.

F. umbilicata JCM 30896^T^ was grown on Eggerth-Gagnon agar (Merck) supplemented with 5% (vol/vol) horse blood for 4 days at 37°C under a H_2_-CO_2_-N_2_ (1:1:8, by volume) gas mixture. Total genomic DNA was extracted from F. umbilicata JCM 30896^T^ using a Genomic-tip 100/G kit (Qiagen). Labiase (5.0 mg/ml; Cosmo Bio) was used to lyse bacterial cells. A whole-genome shotgun library was constructed using a SMRTbell template prep kit 1.0 (Pacific Biosciences), followed by single-molecule real-time (SMRT) sequencing conducted on the PacBio RS II sequencing system (Pacific Biosciences) by Takara. A total of 97,745 reads (311-fold coverage) with an average length of 13,372 bp were assembled *de n*ovo using Hierarchical Genome Assembly Process version 3.0 (HGAP3.0) in SMRT Analysis version 2.3.0 (4), resulting in 8 contigs with an *N*_50_ value of 1,731,485 bp. The default settings for genome assembly were used. This assembly resulted in a draft genome sequence of 3,262,821 bp with a G+C content of 41.6%. Analysis of the genome sequences was performed using the DDBJ Fast Annotation and Submission Tool (DFAST; https://dfast.nig.ac.jp/) (5). A total of 3,210 protein-coding sequences, 60 tRNAs, and 18 rRNAs were detected.

The genome of F. umbilicata JCM 30896^T^ contained genes involved in acetate synthesis, such as formate acetyltransferase (EC 2.3.1.54), phosphotransacetylase (EC 2.3.1.8), and acetate kinase (EC 2.7.2.1). In addition, F. umbilicata JCM 30896^T^ was predicted to possess a vitamin B_12_ biosynthesis pathway. Vitamin B_12_ is an important cofactor for a variety of enzymes. In the systematic genome assessment of B vitamin biosynthesis, vitamin B_12_ biosynthesis was present in 42% of the genomes of 256 common human gut bacteria (6). Furthermore, nearly half of the *Firmicutes* genomes (43%; 56/130) were predicted to synthesize vitamin B_12_ (6). Recently, true bidirectional metabolic cross-feeding dependent on vitamin B_12_ was observed between the mucin-degrading bacterium Akkermansia muciniphila and the butyrate-producing bacterium Eubacterium hallii (7). The production of vitamin B_12_ and acetate by F. umbilicata might be beneficial for growth with other bacteria lacking the vitamin B_12_ biosynthesis pathway, such as A. muciniphila (8), and production of butyrate by butyrate-producing bacteria, such as F. prausnitzii.

The genome sequence will facilitate further studies of the beneficial role of this strain in the human gut.

### Data availability.

This whole-genome shotgun project has been deposited in DDBJ/ENA/GenBank under the accession no. BHEO00000000. The version described in this paper is the first version, BHEO01000000.
